# Identification of a botanical inhibitor of intestinal diacylglyceride acyltransferase 1 activity via *in vitro* screening and a parallel, randomized, blinded, placebo-controlled clinical trial

**DOI:** 10.1186/s12986-015-0025-2

**Published:** 2015-08-06

**Authors:** Rodney A. Velliquette, Kerry Grann, Stephen R. Missler, Jennifer Patterson, Chun Hu, Kevin W. Gellenbeck, Jeffrey D. Scholten, R. Keith Randolph

**Affiliations:** Department of Analytical Sciences, Amway R&D, 7575 Fulton St., Building 50-2D, Ada, MI 49355 USA; Nutrition Product Development, Food, Beverages and Chewables, Amway R&D, Ada, MI 49355 USA; Nutrition Product Development, Supplements, Nutrilite Health Institute, Buena Park, CA 90622 USA

## Abstract

**Background:**

Diacylglyceride acyltransferase 1 (DGAT1) is the enzyme that adds the final fatty acid on to a diacylglyceride during triglyceride (TG) synthesis. DGAT1 plays a key role in the repackaging of dietary TG into circulating TG rich chylomicrons. A growing amount of research has indicated that an exaggerated postprandial circulating TG level is a risk indicator for cardiovascular and metabolic disorders. The aim of this research was to identify a botanical extract that inhibits intestinal DGAT1 activity and attenuates postprandial hypertriglyceridemia in overweight and obese humans.

**Methods:**

Twenty individual phytochemicals and an internal proprietary botanical extract library were screened with a primary cell-free DGAT1 enzyme assay that contained dioleoyl glycerol and palmitoleoyl Coenzyme A as substrates plus human intestinal microsomes as the DGAT1 enzyme source. Botanical extracts with IC50 values < 100 μg/mL were evaluated in a cellular DGAT1 assay. The cellular DGAT1 assay comprised the analysis of ^14^C labeled TG synthesis in cells incubated with ^14^C-glycerol and 0.3 mM oleic acid. Lead botanical extracts were then evaluated in a parallel, double-blind, placebo-controlled clinical trial. Ninety healthy, overweight and obese participants were randomized to receive 2 g daily of placebo or individual botanical extracts (the investigational product) for seven days. Serum TG levels were measured before and after consuming a high fat meal (HFM) challenge (0.354 L drink/shake; 77 g fat, 25 g carbohydrate and 9 g protein) as a marker of intestinal DGAT1 enzyme activity.

**Results:**

Phenolic acids (i.e., gallic acid) and polyphenols (i.e., cyanidin) abundantly found in nature appeared to inhibit DGAT1 enzyme activity *in vitro*. Four polyphenolic rich botanical extracts were identified from *in vitro* evaluation in both cell-free and cellular model systems: apple peel extract (APE), grape extract (GE), red raspberry leaf extract (RLE) and apricot/nectarine extract (ANE) (IC50 = 1.4, 5.6, and 10.4 and 3.4 μg/mL, respectively). In the seven day clinical trial, compared to placebo, only GE significantly reduced the baseline subtracted change in serum TG AUC following consumption of the HFM (AUC = 281 ± 37 vs. 181 ± 30 mg/dL*h, respectively; *P* = 0.021). Chromatographic characterization of the GE revealed a large number of closely eluting components containing proanthocyanidins, catechins, anthocyanins and their secondary metabolites that corresponded with the observed DGAT1 enzyme inhibition in the cell-free model.

**Conclusion:**

These data suggest that a dietary GE has the potential to attenuate postprandial hypertriglyceridemia in part by the inhibition of intestinal DGAT1 enzyme activity without intolerable side effects.

**Trial registration:**

This trial was registered with ClinicalTrials.gov NCT02333461

**Electronic supplementary material:**

The online version of this article (doi:10.1186/s12986-015-0025-2) contains supplementary material, which is available to authorized users.

## Background

Overweight and obese conditions develop as a result of chronic energy imbalance, arising when energy input exceeds energy expenditure. Sedentary lifestyle and the availability of inexpensive, highly palatable, energy-dense foods that are high in fat and refined carbohydrates are major drivers of the global obesity epidemic [1]. These foods displace healthier options, such as fruits and vegetables, from the diet and promote storage of excess calories as body fat. Fruits and vegetables have long been known to be important sources of vitamins, minerals and fiber and are increasingly recognized for the complex and diverse collection of health-promoting phytochemicals they contribute to the diet [2].

Elevated blood triglyceride (TG), another consequence of an eating pattern that is high in fat and refined carbohydrate, is recognized as an independent risk factor for conditions such as metabolic syndrome, type II diabetes and cardiovascular disease [3]. In addition, there is growing evidence that postprandial TG levels strengthens the assessment of metabolic and cardiovascular risk [4–7]. Zilversmit [8] first reported that postprandial hyperlipidemia could be a significant metabolic factor contributing to the development of atherogenesis. He proposed that postprandial TG rich chylomicrons are as atherogenic as circulating low-density lipoprotein cholesterol (LDL-C), and emphasized the need for studies that address this phenomenon. Research around postprandial hypertriglyceridemia continues to be a topic of great interest [4–7, 9–12]. In fact, postprandial TG levels have been reported to be more predictive of cardiovascular risk compared to fasting TG [9, 13]. The circulating TG levels in response to a meal are dependent on many variables including total amount and type of dietary fats, fasting TG levels, gastric emptying, intestinal break down, absorption and secretion of TG and systemic catabolism. Given humans spend a significant amount of time in the postprandial state, the chronic systemic exposure of postprandial hypertriglyceridemia could contribute to multiple metabolic disorders via multiple mechanisms [14–21]. Therefore, nutritional approaches that target postprandial hypertriglyceridemia in response to a fatty meal is mechanism that could have meaningful impact on cardiovascular and metabolic risks and health.

**Fig. 1:**
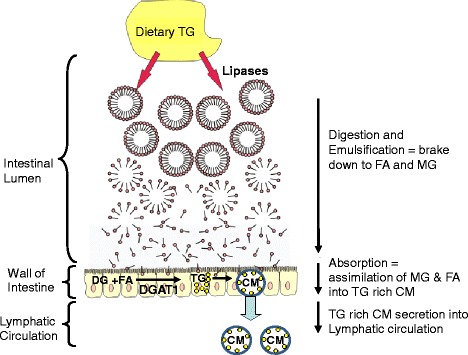
Role of DGAT1 in the assimilation of dietary TG into circulating TG rich chylomicrons (CM). Dietary TG are first broken down into monoacylglycerides (MG) and fatty acids (FA) by a host of pancreatic lipases. MG and FA are then absorbed into the small intestinal enterocytes and repackaged into diacylglyercides (DG) by monoacylglyceride acyltransferase transferase. DGAT1 then acylates the DG into TG (yellow circles), which are incorporated into CM and secreted into the lymphatic circulation then enter the blood circulation via the thoracic duct

Diacylglycerol acyltransferase 1 (DGAT1) in enterocytes is a key enzyme involved in the assembly of TG from dietary fatty acids [22]. In the postprandial state, intestinal DGAT1 generated TG are secreted into the lymphatic system, mainly as chylomicron particles, and then enter the blood circulation via the thoracic duct (Fig. 1). DGAT1-deficient (Dgat1−/−) mice are resistant to high fat diet-induced obesity due in part to an increase in systemic energy expenditure induced by body heat loss [23]. It was later reported that intestinal only DGAT1 deficiency could reproduce many of the high fat diet induced phenotypes of the Dgat1−/− mouse [24]. In addition, intestinal only expression of DGAT1 abolished the anti-obesity phenotypes of Dgat1−/− mouse while on a high fat diet [25]. These animal studies lead to the pharmaceutical development of intestinal DGAT1 inhibitors as a mechanistic approach to mitigate metabolic risks associated with elevated postprandial TG levels [12, 26, 27]. These and other reports suggest that delaying and decreasing postprandial circulating TG levels with intestinal DGAT1 inhibition could facilitate the improvement of metabolic and cardiovascular risks and maintenance of health [22, 24, 28–31]. However, a hurdle facing pharmaceutical inhibitors thus far has been the compounds are so selective and potent that the gastrointestinal side effect profiles, like nausea, diarrhea and vomiting are not tolerated [12]. Therefore, nutritional strategies targeting postprandial TG levels via DGAT1 inhibition without intolerable side effects could have meaningful impact on cardiovascular and metabolic risks and health.

We are not aware of any published prospective human studies that provide evidence for a botanical inhibitor of intestinal DGAT1 activity indicated by measuring serum TG during a high fat meal (HFM). The aim of this research was to identify a botanical extract that inhibits intestinal DGAT1 activity and attenuates postprandial hypertriglyceridemia in overweight and obese humans. This report presents *in vitro* and *in vivo* evidence supporting inhibition of intestinal DGAT1 activity via a botanical extract that may have the potential to improve metabolic imbalance related to postprandial hypertriglyceridemia.

## Methods

### Clinical trial methodology

The randomized, double-blind, placebo-controlled clinical trial was approved by the New England Institutional Review Board (Wellesley, MA, USA) and was conducted in compliance with the Declaration of Helsinki and the International Conference on Harmonization Guidelines. Informed consent to participate in the research study was obtained from all study participants using an IRB-approved consent form (NEIRB # 12–103). The study was conducted from May through August, 2012 at Radiant Research, Chicago, IL.

A total of 158 healthy overweight and obese individuals were screened based on medical history, vital signs, physical exam, height/weight, concomitant medication use, serum chemistry, hematology and lipid panel as well as a urine pregnancy test for females of childbearing age. Participants were excluded if they had a fasting TG > 200 mg/dL or any abnormal biochemical value deemed to be clinically significant by the Principal Investigator. Women were excluded if they were pregnant or lactating or were of child bearing age and unwilling to use birth control. The exclusion criteria included an alcoholic beverage intake > 14 drinks per week and history of gastric bypass or other surgery to physically alter the gastrointestinal tract. Concomitant use of medications for blood pressure, coagulation disorders, high cholesterol, gastroesophageal reflux, or any medication with vasoconstricting properties such as serotonin reuptake inhibitors or monoamine oxidase inhibitors was an additional exclusion criteria.

Subjects were also excluded if they had consumed antibiotics in the previous week, dietary supplements (e.g. vitamins, minerals and herbal products, including herbal drinks) within one week, fish oil supplements or consumption of fatty fish more than once per week within eight weeks of the trial. Those who were eligible to participate were instructed to maintain their normal diet and physical activity pattern throughout the duration of the study.

### Investigational product (Botanical Extracts)

The investigational product doses were packed in opaque two piece hard shell capsules. Each capsule had a fill weight of 333 mg and contained either 100 % extract or placebo. The placebo was comprised of silicified microcrystalline cellulose, magnesium stearate, modified cellulose gum, silicon dioxide, dextrose, corn starch and caramel color. Each qualified participant received one bottle of investigational product that contained placebo, apple peel extract (APE), grape extract (GE), red raspberry leaf extract (RLE) or apricot/nectarine extract (ANE). See Table 1 for botanical extract details. Participants were instructed to ingest the investigational product as six capsules once daily (total of 2 g/d) with the morning meal for seven days. Participants returned their bottle of investigational product at the final study visit.

**Table 1:** Botanical extract information

Extract	Manufacturer	*Genus/Species*	Plant Part	Standardization	Extraction Solvent	Extraction Ratio	Excipient
Apple Peel	Cyvex Irvine, CA	*Malus Domestica*	Skin	80 % Polyphenol, 5 % Phlorizin	Ethanol/Water	115:1	None
Grape	Cyvex Irvine, CA	*Vitus Vinifera*	Pulp, Skin, Seed	75 % Total Polyphenol, 50 % Oligomeric Proanthocyanidin	Ethanol/Water	8000:1	None
Red Raspberry Leaf	Naturex South Hackensack, NJ	*Rubus Idaeus*	Leaf	6 % Ellagic Acid	Ethanol/Water	4:1	Maltodextrin, Silica
Apricot/Nectarine	PLT Health Morristown, NJ	*Prunus Armeniaca, Prunus Persica*	Whole Fruit	50 % Polyphenol	Ethanol/Water	40:1	None

### Clinical trial procedures

Ninety participants were randomly assigned in blocks of five to one of the five test groups. The test groups within each block were allocated using a random number generator (Statistical Analysis Program, Cary, NC). The randomization codes were kept at a separate location in the Quality Assurance Department of Radiant Development.

Participants were instructed to fast for 12 h and refrain from strenuous physical activity the morning of each study day (Day 1 and 7). A standardized high fat meal (HFM) drink/shake containing 77 g fat, 25 g carbohydrate, and 9 g protein (Table 2) was administered over a 15 min period at baseline (day 1) and following one week (day 7) of placebo or investigational product. The HFM composition was similar to the recommendations reported by Kolovou *et al*. [32]. At the final study day (day 7), the final dose of the placebo or investigational product was consumed 10 min before the time zero blood draw and consumption of the HFM. On both study days, blood was drawn via standard venipuncture immediately before and 2, 4, and 6 h after consuming the HFM.

**Table 2:** Recipe of the HFM

	Fat (g)	Carbohydrate (g)	Protein (g)	Energy (kcal)
Dean’s Whipping Cream	66	0	0	594
Eddy’s Grand Vanilla Ice Cream	11	24	3	202
Amazing Egg	0	1	6	28
Total	77	25	9	824

All ingredients were placed in a blender and mixed. Fat = 83.5 %; Carbohydrate = 12.0 %; Protein = 4.4 %. Each shake = 0.354 L (12 oz)

**Fig. 2:**
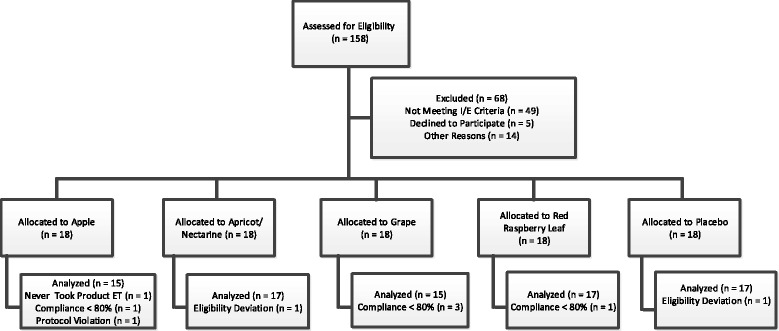
Clinical trial participant flow

Compliance was evaluated by participant interview and counting the investigational product capsules returned to the clinic at final study day. Non-compliance was defined as consumption of <80 % of the scheduled intake of investigational product. Due to the short intervention period, participants with compliance below this threshold were removed from the study efficacy analysis. Figure 2 details the clinical trial participant flow.

Safety and tolerability were assessed by measuring changes in serum chemistry, hematology, vital signs, body weight and reported adverse events.

### Outcome variables

The primary outcome variable was serum TG levels over a 6 h time period following consumption of a standardized HFM. Serum total cholesterol (TC), high-density lipoprotein cholesterol (HDL-C) and TG were measured on the Siemens ADVIA 1800, and low-density lipoprotein cholesterol (LDL-C) was calculated by the Friedewald equation: LDL-C = (Total Cholesterol) – (HDL-C) – (very low-density lipoprotein (triglycerides/5)) [33].

### Cell-free DGAT1 assay

A cell-free DGAT1 assay was established using a human small intestinal microsomal preparation (BD Biosciences) as the enzyme source, and dioleoyl glycerol and palmitoleoyl Coenzyme A (CoA) as substrates (Sigma). Dioleoyl glycerol was dissolved in DMSO and diluted to 600 μM with 175 mM Tris–HCl and 100 mM MgCl_2_ (Tris/MgCl_2_) buffer. Palmitoleoyl CoA was dissolved in1.5 % acetone water solution (Fisher Scientific) to 150 μM. Microsomes were diluted to 25 μg/mL with 3.5 mg/mL BSA in Tris MgCl_2_ buffer. To a 1.5 mL tube, 67 μL of each palmitoleoyl CoA s and dioleoyl glycerol solutions (1:1 ratio) were added and the reaction was initiated by the addition of 67 μL of microsomes. The reaction was incubated in a 37 °C water bath for 60 min. The reaction was stopped by the addition of 1 mL of extraction solvent (isopropanol:dichloromethane (1:1 v/v) with 2.4 % formic acid) containing 1 μg/mL internal standard (glyceryl triolein, Sigma) to each tube and mixed by vortexing. The tubes were centrifuged at 10,000Xg for 3 min. 500 μL of the bottom phase was filtered through a Millex-HV syringe driven filter unit with tube outlet (PVDF @ 0.45 μm, Millipore) and transferred to LC-MS vials (12x32 mm glass screw neck, preslit cap & PTFE/silicone septa, Waters). Phytochemicals (Sigma), botanical extracts and positive control were diluted in DMSO and added to the reaction with the substrates at the desired concentration. The DGAT1 inhibitor A922500 (Tocris) was used as positive control at a concentration of 40 nM.

### LC-MS analysis

Refer to Table 3 for the mobile phase program. Solvent A: 0.1 % ammonium acetate in water; Solvent B: 0.1 % acetic acid in acetonitrile; Solvent C: 0.1 % acetic acid in isopropanol; all solvents were from Fisher Scientific. Injection volume: 10 μL. Column: Acquity BEH C18 1.7 μm, 2.1 x 50 mm. Effluent from the column was directed to the electrospray source of the mass spectrometer. Electrospray conditions: positive ion mode; cone voltage: 50 eV; desolvation temperature: 450 °C; source temperature: 140 °C; nebulizer gas flow: 800 L/h; cone gas flow: 10 L/h. The mass spectrometer was operated at 20,000 resolving power and scans from m/z 100–1200 were acquired with a cycle time of 0.25 s. Selected ion chromatograms were plotted for the following: m/z 875 representing the ammoniated molecular ion for triacylglycerol reaction product containing 2 oleic acid and 1 palmitoleic acid moieties (C18:1/C18:1/C16:1); m/z 903 for the ammoniated molecular ion of glyceryl triolein (C18:1/C18:1/C18:1) internal standard. Quantification was determined based on area counts of the product relative to the internal standard.

**Table 3:** Liquid chromatography mobile phase program

Time (min)	Flow rate (mL/min)	Solvent A (%)	Solvent B (%)	Solvent C (%)
0	0.4	50	50	0
5	0.4	0	50	50
7	0.4	0	50	50
8	0.4	50	50	0
10	0.4	50	50	0

### Cellular DGAT1 assay

Three different cell lines, human colorectal (HT-29), human embryonic kidney (HEK293H) and human hepatic (HEPG2) were screened for the presence of both DGAT1 and DGAT2 protein. The HEK293H cell line contained DGAT1 and little to no DGAT2 protein (Additional file 1: Figure S1), therefore, a cellular DGAT1 assay was established in HEK293H cells to measure the synthesis of TG. HEK293 cell line has previously been reported to contain only DGAT1 and not DGAT2, and has been validated as an *in vitro* model to determine TG synthesis via DGAT1 [34].

The DGAT1 activity was determined by measuring the incorporation of [^14^C]-glycerol (1 μCi/mL, Perkin-Elmer) into [^14^C]-TG during a 5 h incubation with 0.3 mM oleic acid/BSA in HEK293H cells. Cells were plated in 12-well culture plates at 45,000/cm^2^, high glucose Dulbecco modified eagle medium (DMEM) supplemented with 10 % (v/v) fetal bovine serum (HyClone), 100 U/mL penicillin and 100 μg/mL streptomycin (Life Technologies) and allowed to adhere overnight in a humidified atmosphere with 5 % CO_2_ at 37 °C. Medium was then changed to serum-free DMEM, and vehicle, positive control (A922500) or botanical extracts were added and pre-incubated with the cells for 30 min prior to stimulating TG synthesis with oleic acid. After 5 h of treatment, cells were harvested with cold phosphate buffered saline. Cellular lipids were then extracted with chloroform:methanol (1:2 v/v) and centrifuged. The upper phase was aspirated and the organic phase was dried under nitrogen. Lipids were solubilized with a small amount of chloroform containing a TG standard (Perkin-Elmer) and separated by TLC using toluene/chloroform/methanol mobile phase. Lipid species were identified by iodine vapor and compared to standards. Silica gel spots corresponding to lipid species were scraped into scintillation vials and the incorporated radioactivity was quantified using a MicroBeta TriLux (Perkin-Elmer). All cellular, radiolabeled DGAT1 assay experiments were conducted at Zen-bio Inc. (Research Triangle Park, NC).

### Bioassay directed fractionation (BDF) of GE

GE was prepared at a concentration of 50 mg/mL in Optima™ LCMS grade water and filtered using a Whatman 25 mm, 0.45 micron GD/X syringe filter (Fisher Scientific, Pittsburgh, PA) into 1.5 mL HPLC autosampler vials for analysis. The injection volume was 10 μL/run, providing 500 μg of GE extract per injection. Chromatographic separation of phytochemicals in the diluted extract was performed on a Acquity UPLC-H chromatograph equipped with a photodiode array detector (PDA) and XBridge Shield RP18 (5 μm, 4.6 x 250 mm) column (Waters Corp, Milford, MA). The mobile phase solutions used for gradient separation were prepared with Optima™ LCMS grade solvents (Fisher Scientific, Pittsburgh, PA) as follows: A, 0.1 % acetic acid in water and B, 0.1 % acetic acid in acetonitrile. The mobile phase gradient (A:B), at ambient temperature and a flow rate of 0.8 mL/min, was initially set at 95:5 and linearly changed to 0:100 from 0 to 30 min, held for 2 min, and then returned to initial conditions at 32.1 min and held until 35 min. UV-visible data were acquired from 210 – 800 nm at a 1.2 nm resolution using a Waters Acquity PDA eLambda detector.

The effluent was split 10:1 after the PDA detector, with the majority going to a fraction collector configured for 96 well plates with 2 mL well volume. Fractions were collected at 20 s intervals for 32 min. A total of 4 injections per collection plate was performed, providing approximately 1.8 mg of GE extract per plate. The plates were frozen at −80 °C overnight, and the solvent subsequently removed by freeze drying. The dried sample plates were sealed and stored dry at −20 °C until assayed in the cell-free DGAT1 assay.

The remaining effluent was directed to a Synapt G2 QTOF mass spectrometer (Waters Corp, Milford, MA) equipped with an electrospray ion source and operated at 25 V cone voltage. Accurate mass spectra were collected in separate runs for both positive and negative ions, using Leu-enkephalin as a mass marker. Data were collected from m/z 100–1200 at 0.5 s/spectrum. Alternating spectra were collected at 0 eV and 20 eV collision energy per cycle, with argon used as the collision gas in the transfer cell (MS^*e*^ mode). Mass spectra, UV spectra and bioassay responses for fractions were time aligned for identification of phytochemicals corresponding to the observed DGAT1 enzyme inhibition.

### Statistical methods

Dose response curves for the cell-free DGAT1 assay were fitted with a log (inhibitor) vs. response, variable slope (four parameters) equation. Cellular DGAT1 assay data is shown as mean ± standard error of mean (SEM) and was analyzed by two-way ANOVA with Bonferroni's multiple comparison test. The primary outcome variable, serum TG level, is presented as mean ± SEM of baseline (fasting) subtracted change in serum TG after the HFM challenge. Area under the curve (AUC) was determined using the trapezoidal method. Comparisons between groups were made using unpaired *t*-test with Welch’s correction. A significant effect of investigational product was defined as *P* < 0.05. All statistical analyses were done using Prism-Graph Pad Software (San Diego, CA).

## Results

### *In vitro* DGAT1 screening

**Fig. 3:**
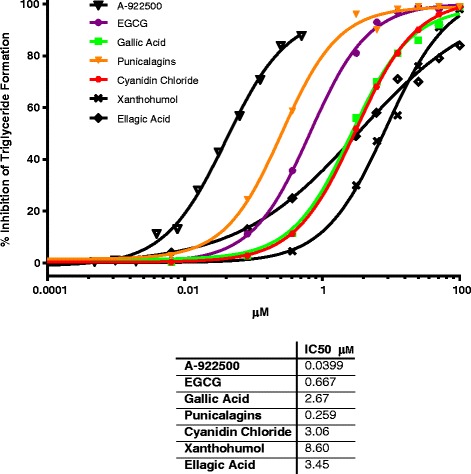
Percent DGAT1 enzyme inhibition and IC50 values for single phytochemicals in the cell-free assay. Twenty individual phytochemicals were screened through the cell-free DGAT1 enzyme assay. Six of the twenty phytochemicals were dose dependent inhibitors of DGAT1 enzyme activity (IC50 ranged from 0.667 to 8.60 μM, compared to A-922500 = 40nM) and all were phenolic acids or polyphenols. Results are the mean of duplicates

We first screened a panel of 20 phytochemicals, mostly polyphenols and phenolic acids (Apigenin, Astilbin, Catechin, Cyanidin Chloride, Dihydrokaempferol, Epigallocatechin Gallate (EGCG), Ellagic Acid, Formononetin, Gallic Acid, Guggulsterone, Kaempferol, Icariin, Luteolin, Naringenin, Paeoniflorin, Punicalagins, Orientin, Sparteine, Taxifolin and *trans*-Resveratrol) in the cell-free DGAT1 assay to aid in the selection of prospective botanical extracts for screening. Phytochemicals with ≥50 % DGAT1 inhibition at 50 μM were further examined in titration experiments. Six phytochemicals were dose dependent inhibitors of DGAT1 enzyme activity (Fig. 3) and all were polyphenols or phenolic acids (data not shown for remaining fourteen phytochemicals). The IC50 values ranged from 0.667 to 8.60 μM compared to 39.9 nM for the synthetic inhibitor A922500.

**Fig. 4:**
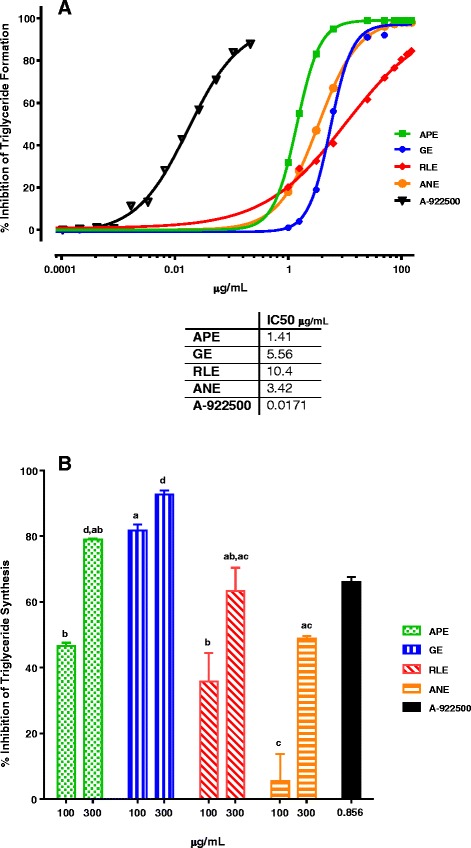
Percent DGAT1 enzyme inhibition and IC50 values for the four lead botanical extracts. **a** APE, GE, RLE and ANE exhibited dose responsive inhibition of DGAT1 enzyme activity in the cell-free assay. The IC50 values ranged from 1.41 to 10.4 μg/mL and 17.1 ng/mL for A-922500. Results are the mean of triplicates. **b** The cellular DGAT1 assay was comprised of adding [^14^C]-glycerol to label newly synthesized TG and 0.3 mM oleic acid/BSA to stimulate DGAT1 activity. All botanical extracts and A-922500 inhibited oleic acid induced DGAT1 enzyme activity as measured by ^14^C label TG levels. GE was statistically more potent than APE, RLE and ANE, defined as greatest inhibition at the lowest dose (100 μg/mL) (Two-way ANOVA with Bonferroni's multiple comparisons test; *P* < 0.001). Result are the mean ± SEM (*n* = 3). Different letters, within each dose, indicate statistically significant

Botanical extracts from an internal, proprietary botanical extract library were selected based on their predicted presence of the polyphenolic inhibitors presented in Fig. 3, and screened through the cell-free DGAT1 assay. Four lead botanical extracts emerged from the library screening (Fig. 4): 1) apple peel extract (APE); 2) grape extract (GE); 3) red raspberry leaf extract (RLE); 4) apricot/nectarine extract (ANE) with IC50 values in cell-free assay of 1.4, 5.6, 10.4 and 3.4 μg/mL, respectively (Fig. 4a). In the cellular DGAT1 assay, all extracts inhibited TG synthesis ≥ 50 % at 100 and/or 300 μg/mL. While all botanical extracts were effective in this cellular assay, the GE was the most potent (greatest inhibition at the lowest dose) with APE and RLE having equally potency, and ANE the least. (Fig. 4b, *P* < 0.001).

### Clinical trial

Participant flow through the study protocol is outlined in Fig. 2. Ninety participants were randomized into 5 test groups: Placebo, APE, GE, RLE and ANE (*n* = 18/group). Eighty-one participants completed the trial per protocol and Table 4 shows the day 1 characteristics of these participants. There were no significant differences in age, body mass index (BMI) or fasting, TC, LDL-C, HDL-C or TG at baseline between groups. Gender was not balanced across the test groups.

**Table 4:** Day 1 and day 7 characteristics of participants completing the trial per protocol

Characteristics	Test Day	Placebo	APE	GE	RLE	ANE
N (Male/Female)		17 (9/8)	15 (7/8)	15 (6/9)	17 (6/11)	17 (11/6)
Age (years)		46.7 ± 9.9	47.0 ± 9.3	52.7 ± 12.6	46.8 ± 8.2	48.4 ± 9.0
Body Weight (kg)	1	90.8 ± 14.7	91.0 ± 10.4	88.3 ± 15.1	86.3 ± 13.6	87.9 ± 9.7
	7	90.8 ± 14.7	91.1 ± 10.7	88.0 ± 15.3	86.3 ± 13.6	88.0 ± 9.6
BMI	1	30.2 ± 3.9	32.0 ± 1.8	30.8 ± 2.9	30.8 ± 3.5	30.4 ± 3.5
	7	30.1 ± 3.9	32.0 ± 1.9	30.7 ± 3.0	30.5 ± 3.4	30.4 ± 3.5
Total Cholesterol (mg/dL)	1	206 ± 41	183 ± 30	190 ± 46	196 ± 34	200 ± 46
	7	195 ± 44	170 ± 25	175 ± 35	191 ± 31	184 ± 35
LDL-C (mg/dL)	1	135 ± 42	116 ± 30	115 ± 42	123 ± 34	133 ± 43
	7	129 ± 43	107 ± 25	105 ± 29	117 ± 34	119 ± 35
HDL-C (mg/dL)	1	50.3 ± 18.1	51.0 ± 17.2	58.7 ± 17.2	57.8 ± 17.7	50.1 ± 12.5
	7	48.2 ± 19.4	46.3 ± 13.5	55.4 ± 19.8	54.8 ± 19.2	46.8 ± 11.2
TG (mg/dL)	1	97.5 ± 39.8	96.7 ± 39.8	101.7 ± 38.7	102.3 ± 35.8	111.1 ± 46.8
	7	95.8 ± 36.2	94.7 ± 26.4	90.3 ± 23.8	102.1 ± 57	103.8 ± 40.4

There were no statistically significant differences in any of the measured anthropometric or serum lipids between groups at day 1 or at day 7. Results are Mean ± SD

No placebo or investigational product related changes were observed in vital signs, serum chemistry or hematology parameters (data not shown). Nineteen study participants experienced an adverse event (AE) considered to be potentially related to placebo or investigational product. All of these AE were graded as mild and did not impact study participation (Additional file 2: Table S1). Diarrhea, potentially related to fat malabsorption, was not reported by any subject.

**Fig. 5:**
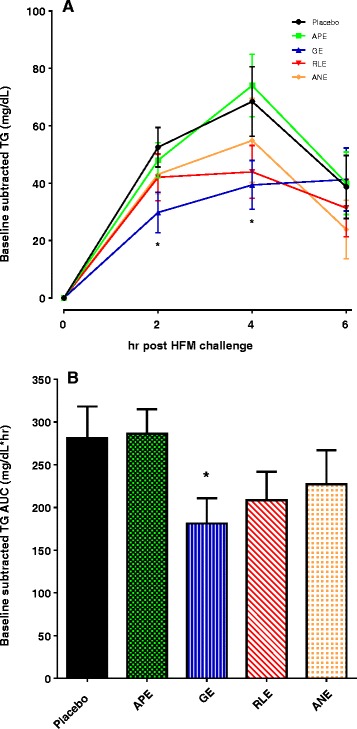
Baseline (fasting levels) subtracted change in serum TG levels following a HFM challenge after seven days of placebo or investigational product. **a** Baseline subtracted serum TG response over a 6 h period. GE significantly reduced the serum TG levels at 2 h (*p* = 0.014) and 4 h (*p* = (0.029) after the HFM compared to placebo. **b** AUC for the baseline subtracted serum TG was also significantly reduced by seven days of dietary GE (*p* = 0.021). Fasting serum TG = 95.8 ± 8.8; 94.7 ± 6.8; 90.3 ± 6.2; 102.1 ± 13.8; 103.8 ± 9.8 mg/dL, for placebo, APE, GE, RLE and ANE, respectively. Results are mean ± SEM. Comparisons between placebo and investigational product test groups were made using unpaired *t*-test (* *P* < 0.05)

There was no significant difference across all groups in the baseline (fasting) subtracted change in serum TG AUC after the HFM challenge at day 1 (data not shown). After seven days of placebo or investigational products, only the GE significantly impacted the baseline subtracted change in serum TG levels following the HFM challenge compared to placebo. GE significantly reduced the 2 h (*P* = 0.014) and 4 h (*P* = 0.029) baseline subtracted change in serum TG compared to placebo (Fig. 5a). This translated into a significant reduction in the AUC (Fig. 5b; *P* = 0.021). No significant differences in fasting TC, LDL-C, HDL-C or TG were seen after seven days of placebo or investigational product (Table 4).

### Bioassay-directed fractionation (BDF) of GE

**Fig. 6:**
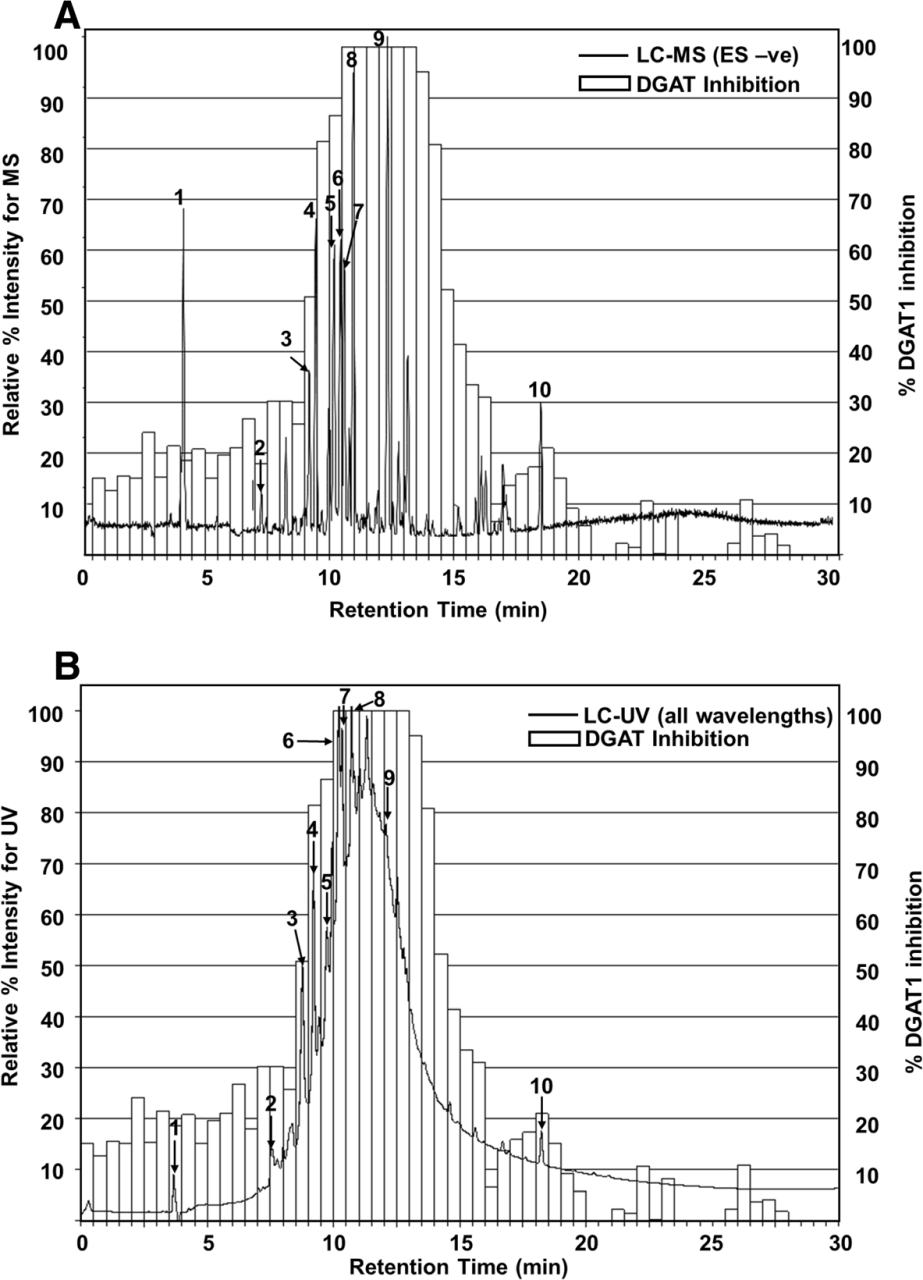
Cell-free DGAT1 inhibition from chromatographic fractionation of GE with overlay of the corresponding LC-MS and LC-UV chromatograms. **a** The LC-MS chromatogram was generated using base peak index utility to differentiate major compounds. **b** The LC-UV chromatogram represents the sum of all wavelengths to better show the complex mixture and abundance of phytochemicals in the GE extract. DGAT1 enzyme inhibition (right side x-axis) correlates with the LC-UV pattern and abundance (left side x-axis), showing that multiple phytochemicals in the GE extract are responsible for the observed inhibition. Identities of numbered peaks are given in Table 5

In efforts to identify potential bioactive phytochemicals from GE, we chromatographically fractionated GE into a 96-well plate. Fractions were subsequently screened through the cell-free DGAT1 enzyme assay. Chromatographic characterization of GE revealed a large number of closely eluting components containing primarily proanthocyanidins, catechins, anthocyanins and other polyphenols based on mass spectral and UV-visible data (Fig. 6a, b). The relative amount of DGAT1 enzyme inhibition (right side X-axis) for the fractions paralleled chromatographic abundance of components in the GE (left side X-axis). These data suggest that there are multiple polyphenols in GE that could be contributing to the inhibition of DGAT1 enzyme activity. Table 5 lists ten polyphenols that were identified based on accurate mass and UV–vis spectra data. Peaks 3–9 (malvidin-3-glucoside, procyanidin B1, catechin, procyanidin B2, epicatechin, procyanidin C1 and syringic acid, respectively) corresponded in retention time with fractions that inhibited DGAT1 enzyme activity by greater than 50 % in the cell-free assay (Fig. 6a, b). While the phytochemicals co-elute at the time corresponding to the observed DGAT1 enzyme inhibition, they were not necessarily solely responsible for the inhibition of that peak. This is due to the fact there are likely many unresolved and unidentified phenolic acids and polyphenols that are co-eluting during the 9–16 min retention time frame. Therefore, this unique mixture, and one single phytochemical, is likely responsible of the observed efficacy.

**Table 5:** Phytochemical class and identified peaks from GE

Phytochemical Class	Phytochemical Identity_(peak #)_
Organic Acids	Tartaric acid_(1)_, Gallic acid_(2)_, Syringic acid_(9)_
Catechins	Catechin_(5)_, Epicatechin_(7)_
Oligomeric Proanthocyanidins	Procyanidin B1_(4)_, Procyanidin B2_(6)_, Procyanidin -C1_(8)_
Anthocyanins	Malvidin-3-glucoside_(3)_
Flavones	Quercetin_(10)_

## Discussion

The circulating TG levels in response to a HFM challenge has been validated in both animal and human studies to be a method of examining intestinal DGAT1 activity [25, 27, 35–38]. Here we report the first demonstration in *vitro* and in humans that a GE inhibits intestinal DGAT1 as measured by the change in serum TG following a HFM challenge. Seven days of dietary GE resulted in a significant reduction in the serum TG AUC following the HFM compared to placebo. This effect was unique to GE, as no significant efficacy was observed in the three other investigational products (APE, RLE and ANE) examined in this study.

Intestinal DGAT1 plays a major role in the postprandial TG response by regulating enterocyte TG synthesis and secretion [22, 35, 39]. Intestinal DGAT1 is positioned at a site that makes it a good candidate for impacting the postprandial TG response. Pharmacological inhibitors of DGAT1 have been shown to effectively reduce the postprandial TG response to an HFM challenge [36, 37, 40, 41], but not without significant and intolerable gastrointestinal side effects in humans like nausea, diarrhea and vomiting. This indicates that intestinal DGAT1 is a good target to reduce postprandial TG levels. However, the gastrointestinal side effects due to the high selectivity and potency of pharmaceutical DGAT1 inhibitors has limited their full development. Therefore, if dietary interventions could reduce the chronic systemic exposure of postprandial hypertriglyceridemia by modulating, in part, intestinal DGAT1 activity without significant and intolerable gastrointestinal side effects, this could be a potential dietary strategy to improve multiple cardiovascular and metabolic risks linked to postprandial hypertriglyceridemia.

Grapes in a variety of forms have been reported to provide multiple cardiovascular health benefits [42, 43]. These health benefits have been associated with numerous mechanisms of action [42, 44, 45], and the unique phytochemical groups present in grapes such as simple phenolics, flavonoids, anthocyanins, stilbenes and proanthocyanidin have been reported to be responsible for these health benefits [42, 46–49]. On a broader clarification, all these phytochemical groups belong to the polyphenol class of phytochemicals. Some of these polyphenols are derived from specific components of the grape (i.e., stilbenes in skin, anthocyanins in flesh or proanthocyanidins in seeds) and are the most important class of bioactive compounds in grapes. Grape is one of the richest sources of polyphenols among fruits and over 500 phytochemicals have been identified [50]. Therefore, the wide range of health benefits and pleiotropic effects of grapes and grape products on human health is likely due to the unique phytochemical profile [50–52]. We have identify several of these polyphenols as potential bioactives inhibiting DGAT1 enzyme activity.

The GE used in this study was a highly concentrated extract (8000:1 extraction ratio) of grape pulp, skin and seeds that is 75 % and 50 % by weight polyphenols and oligomeric proanthocyanidins (OPC), respectively (see Table 1). The BDF data suggests there are multiple types of polyphenols (e.g., proanthocyanidins, catechins and anthocyanins) that are contributing to DGAT1 enzyme inhibition (Fig. 6 and Table 5). Interestingly, the APE, RLE and ANE also contain polyphenols, yet no significant *in vivo* efficacy was observed. This is likely due to various factors including the different types, absolute amounts and mutual ratios of polyphenols as they are typically more active when existing as a mixture, rather than in individual forms [53]. Other factors such as intestinal absorption, hydrolysis by small intestine enzymes and/or bacterial modification could have influenced the potential *in vivo* efficacy of the polyphenols [54–56]. Based on the compositional information in Table 5 and our BDF data, it is likely that the unique profile, ratio and amounts of anthocyanin metabolites and OPC in the GE are involved with the observed *in vivo* effect, as these polyphenols are abundant in grapes but not in the other three botanical extracts.

We report several lines of evidence supporting the idea that GE lowers postprandial TG, in part, via inhibition of intestinal DGAT1. To support this MOA, we used a cell-free DGAT1 enzyme screening assay in which DGAT1 activity was derived from human intestinal microsomes, and then employed MgCl_2_ to selectively inhibit any DGAT2 activity present. Secondary confirmation of inhibition was provided by using a cell-based assay in which the cellular model did not express DGAT2 [34]. *In vitro* experiments therefore reflect only inhibition against DGAT1 enzyme activity and not DGAT2. This is relevant since only DGAT1, but not DGAT2 enzyme, has been reported to be present in human small intestine [39, 57, 58]. Finally, clinical assessment of functional activity relied on circulating TG levels in response to a HFM challenge which is a validated model and biomarker to examine intestinal DGAT1 enzyme activity. In this model, pharmacological inhibition of DGAT1 in both rodents and humans results in reduced AUC of circulating TG and increased time to peak plasma TG following a HFM challenge [27, 35, 37, 38, 41]. This postprandial TG profile is similar to results observed in our clinical trial.

It is possible that additional mechanisms and targets could have been affected by the GE that in part contributes to the reduction in the HFM induced hypertriglyceridemia. Mechanisms involved in controlling the rate of gastric emptying may have been impacted via gut derived hormones [59] and/or directly or indirectly by inhibition of intestinal DGAT1 activity [35]. GE may have inhibited other intestinal TG assimilation acyltransferase enzymes (i.e., MGAT1, 2) or metabolic pathways that occur prior to the DGAT1 reaction (i.e., luminal absorption and enterocyte transport). In addition, an effect on chylomicron assembly, transport into the circulation and systemic catabolism cannot be completely ruled out.

## Conclusion

Our research has led to the identification of a dietary GE that inhibits DGAT1 activity *in vitro* and is the first clinical study to show a reduction in postprandial TG response to a HFM in overweight and obese humans at a dose that would be difficult to achieve in the diet with whole grapes. These data suggest that a dietary GE has the potential to provide safe and significant attenuation of high fat diet driven hypertriglyceridemia via a mechanism that may include inhibition of intestinal DGAT1 activity. These results merit further clinical investigation and characterization of bioactive phytochemicals unique to the whole GE. Therefore, we are conducting a longer term clinical study with the GE in a more diverse population and are further characterizing potential bioactives.

## Additional files

Additional file 1: Figure S1. Western blot analysis for the presence of both DGAT1 and DGAT2 protein in human colorectal cell line (HT-29), human embryonic kidney (HEK293H) and human hepatic cell line (HEPG2). (PDF 225 kb) Additional file 2: Table S1. Reported adverse events that were potentially related to placebo or investigation products. (PDF 87 kb)

## Abbreviations

APE: Apple peel extract
ANE: Apricot/Nectarine extract
AUC: Area under the curve
DGAT1: Diacylglyceride acyltransferase 1
GE: Grape extract
HFM: High fat meal
IC50: Inhibition concentration of 50 %
RLE: Red raspberry leaf extract
TG: Triglyceride
