# Supplementary material for: Identification of a botanical inhibitor of intestinal diacylglyceride acyltransferase 1 activity via in vitro screening and a parallel, randomized, blinded, placebo-controlled clinical trial
Source: Nutr Metab (Lond). 2015 Aug 6;12:27. doi: 10.1186/s12986-015-0025-2 (PMC4526202; doi:10.1186/s12986-015-0025-2)
Supplement: Additional file 1: Figure S1. — Western blot analysis for the presence of both DGAT1 and DGAT2 protein in human colorectal cell line (HT-29), human embryonic kidney (HEK293H) and human hepatic cell line (HEPG2). (PDF 225 kb) [file 12986_2015_25_MOESM1_ESM.pdf]

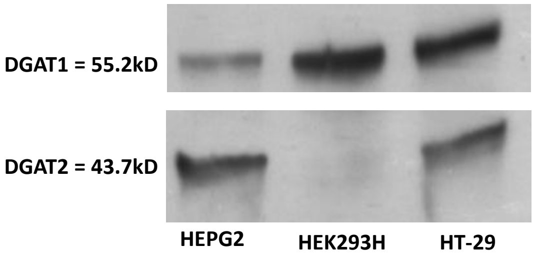

Additional File 1: Figure S1. Western blot analysis for the presence of DGAT1 and DGAT2 in HEPG2, HEK293 and HT-29 cell lines.

Human hepatic cell line (HEPG2), human colorectal cell line (HT-29) and were purchased from ATCC (Manassas, VA) and human embryonic kidney (HEK293H) from Invitrogen (Carlsbad, California). Cells were lysed in RIPA buffer and isolated proteins (20µg) were separated by SDS polyacrylamide gel electrophoresis and transferred to 0.45 µm polyvinylidene difluoride membrane. After transfer, membranes were blocked in 5% non-fat powdered milk in PBS with 0.1% Tween 20 and probed with DGAT1 and DGAT2 antibodies from Santa Cruz Biotechnology (Santa Cruz, CA). Horseradish peroxidase-conjugated secondary antibodies and an enhanced chemiluminescence substrate kit were used in detection of specific proteins (Pierce, Rockford, IL).
