# Supplementary material for: Identification of a botanical inhibitor of intestinal diacylglyceride acyltransferase 1 activity via in vitro screening and a parallel, randomized, blinded, placebo-controlled clinical trial
Source: Nutr Metab (Lond). 2015 Aug 6;12:27. doi: 10.1186/s12986-015-0025-2 (PMC4526202; doi:10.1186/s12986-015-0025-2)
Supplement: Additional file 2: Table S1. — Reported adverse events that were potentially related to placebo or investigation products. (PDF 87 kb) [file 12986_2015_25_MOESM2_ESM.pdf]

Supplement Table 1. Reported adverse events that were potential related to placebo or investigation products.

% OF SUBJECTS PER TEST GROUP	TEST GROUP	REPORTED EVENT	SEVERITY
**5.8**	Placebo	Frequent bowel movements	Mild
**5.8**	Placebo	Nausea	Mild
**5.8**	Placebo	Headache intermittent	Mild
**6.6**	APE	Drowsiness	Mild
**6.6**	APE	Mild anemia	Mild
**6.6**	APE	Mild nausea	Mild
**6.6**	APE	Regular post dosing bowel movements	Mild
**6.6**	APE	Increase energy	Mild
**6.6**	GE	Urticarial rash	Mild
**6.6**	GE	Frequent belching	Mild
**6.6**	GE	Vomiting	Mild
**6.6**	GE	Increased perspiration	Mild
**6.6**	GE	Nausea	Mild
**6.6**	GE	Loss of appetite	Mild
**5.8**	RLE	Loss of appetite	Mild
**5.8**	RLE	Achiness bilateral lower legs	Mild
**5.8**	RLE	Nausea	Mild
**5.8**	RLE	Heartburn	Mild
**5.8**	ANE	Elevated CK	Mild
**5.8**	ANE	Increased flatulence	Mild
